# Supplementary material for: Expression of SARS-Cov-2 Entry Factors in Patients with Chronic Hepatitis
Source: Viruses. 2022 Oct 29;14(11):2397. doi: 10.3390/v14112397 (PMC9699546; doi:10.3390/v14112397)
Supplement: Supplementary file 1 [file viruses-14-02397-s001.zip › viruses-1971728-supplementary.pdf]

Supplementary Tables

Supplementary Table S1. Clinical symptoms due to SARS-Cov-2 infection in patients with chronic hepatitis (*n* = 13) according to etiology.

Symptoms	All patients *n* = 13	Viral hepatitis *n* = 7	NAFLD *n* = 6	*p*-Value
**Respiratory symptoms, *n* (%)**				
Pharyngodynia	2 (15.4%)	-	2 (33.3%)	0.1106
Rhinitis	1 (7.7%)	-	1 (16.7%)	0.2801
Myalgia/Arthralgia	4 (30.8%)	-	4 (66.7%)	0.0126
Fever	7 (53.8%)	3 (42.9%)	4 (66.7%)	0.4095
Coughing	3 (23.1%)	2 (28.6%)	1 (16.7%)	0.6256
Dyspnoea	4 (30.8%)	3 (42.9%)	1 (16.7%)	0.3271
**Gastrointestinal symptoms, *n* (%)**				
Diarrhea	1 (7.7%)	-	1 (16.7%)	0.2801
Anosmia/Ageusia	3 (23.1%)	1 (14.3%)	2 (33.3%)	0.4350
Any symptoms	10 (76.9%)	4 (57.1%)	6 (100%)	0.0790
**Therapy, *n* (%)**				
Any therapy	9 (69.2%)	4 (57.1%)	5 (83.3%)	0.3271
Paracetamol	8 (61.5%)	4 (57.1%)	4 (66.7%)	0.7353
Heparin	2 (15.4%)	1 (14.3%)	1 (16.7%)	0.9093
Steroids	6 (46.1%)	3 (42.9%)	3 (50%)	0.8046
Oxygen therapy	2 (15.4%)	1 (14.3%)	1 (16.7%)	0.9093
Antibiotics	4 (30.8%)	1 (14.3%)	3 (50%)	0.1814
Ordinary hospitalization	2 (15.4%)	1 (14.3%)	1 (16.7%)	0.9093
Intensive care	-	-	-	-

Footnote. NAFLD, non-alcoholic fatty liver disease.

Supplementary Table S2. Histological characteristics of the NAFLD cohort (*n* = 15).

**Histological features**	***n* (%)**
Steatosis	
1	1 (6.6%)
2	7 (46.7%)
3	7 (46.7%)
Lobular inflammation	
0	-
1-2	15 (100%)
Ballooning	
0	-
1-2	15 (100%)
Hepatic fibrosis	
F0-F1	7 (46.7%)
F2	2 (13.3%)
F3-F4	6 (40%)
NAS ≥ 4	14 (93.4%)

Footnote. NAS, non-alcoholic fatty liver disease activity score.

Supplementary Figure S1. Concentration, dimensional distribution and specificity of hepatic EV.

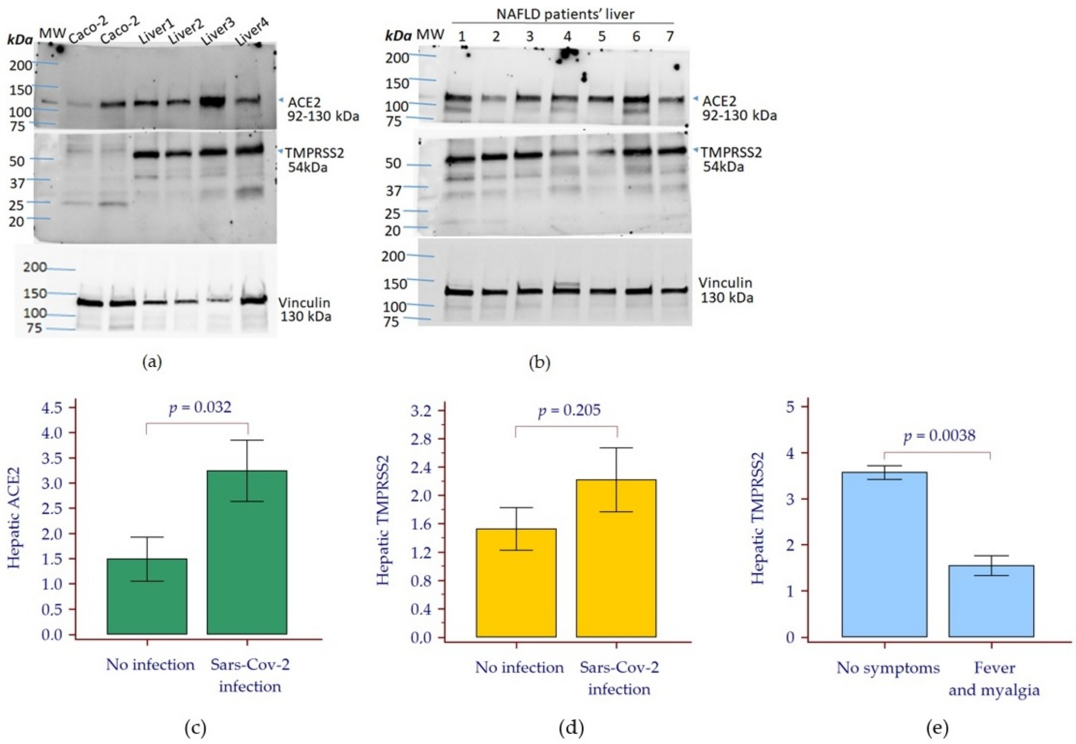

Figure S1. Western blot analysis. Protein extracted from Caco-2 cells were used as positive control and compared to that of liver (samples 1-4)(a). Representative western blot showing expression of ACE2 and TMPRSS2 in liver tissues from 7 NAFLD patients (b). Vinculin was used as housekeeping protein, for data normalization. Hepatic ACE2 (c) and TMPRSS2 (d) levels according to the SARS-Cov-2 infection status. Hepatic TMPRSS2 according to the presence of COVID-19 symptoms (e). ACE2, angiotensin converting enzyme 2; MW, molecular weight marker; SARS-Cov-2, severe acute respiratory coronavirus virus 2; TMPRSS2, transmembrane serine pro-tease 2.
